# Defining linkage to care following human immunodeficiency virus (HIV) diagnosis for public health monitoring in Europe

**DOI:** 10.2807/1560-7917.ES.2018.23.48.1700858

**Published:** 2018-11-29

**Authors:** Sara Croxford, Dorthe Raben, Stine F Jakobsen, Fiona Burns, Andrew Copas, Alison E Brown, Valerie C Delpech, on behalf of OptTEST by HIV in Europe

**Affiliations:** 1 National Infection Service, Public Health England, 61 Colindale Avenue, London, United Kingdom; 2 Institute for Global Health, University College London, Mortimer Market Centre, Capper Street, London, United Kingdom; 3 Center of Excellence for Health, Immunity and Infections, Rigshospitalet, University of Copenhagen, Blegdamsvej 9, Copenhagen, Denmark; 4 Royal Free London NHS Foundation Trust, Pond Street, London, United Kingdom

**Keywords:** human immunodeficiency virus - HIV, epidemiology, surveillance, public health terms

## Abstract

Prompt linkage to human immunodeficiency virus (HIV) care after diagnosis is crucial to ensure optimal patient outcomes. However, few countries monitor this important public health marker and different definitions have been applied, making country and study comparisons difficult. This article presents an expert-agreed, standard definition of linkage to care for a pragmatic approach to public health monitoring, appropriate to the European context. Here, linkage to care is defined as patient entry into specialist HIV care after diagnosis, measured as the time between the HIV diagnosis date and one of the following markers: either the first clinic attendance date, first CD4^+^ cell count or viral load date, or HIV treatment start date, depending on data availability; Linkage is considered prompt if within 3 months of diagnosis. Application of this definition by researchers and public health professionals when reporting surveillance or research data relating to linkage to care after HIV diagnosis will enable reliable comparisons across countries, better assessment of the success of health services programmes aimed at improving peoples access to HIV treatment and care and the identification of barriers limiting access to HIV care across Europe.

## Background

Optimal health outcomes for people with human immunodeficiency virus (HIV) are dependent on diagnosis early in infection, prompt linkage to HIV services after diagnosis, engagement and retention in care and initiation of and adherence to antiretroviral therapy (ART) [1,2]. Late diagnosis of HIV and delays in accessing care and treatment continue to be associated with high mortality rates among people with HIV, particularly in the first year of diagnosis [3].

Public health monitoring of the HIV patient pathway, or continuum of care, is essential to ensure missed opportunities are identified and gaps closed [4]. Research from Europe has focussed on the Joint United Nations Programme on HIV/AIDS (UNAIDS) 90–90–90 targets [5], producing comparable measures for the proportion of people infected with HIV that are diagnosed, on treatment and virally suppressed [6].

However, there are little European data available on linkage to HIV care after diagnosis. A 2014 survey of European countries found there to be little agreement across countries in how to define linkage to care [4]. This lack of consensus is also reflected in the literature from Europe, in which a variety of definitions of linkage to care have been applied, making it difficult to compare across studies [7]. The use of a standard definition would be helpful to more effectively monitor entry into HIV care and facilitate comparisons across countries and populations, target interventions and identify people most at risk of delaying access to care.

In May 2015, the World Health Organization (WHO) released strategic information guidelines to consolidate key indicators to monitor the public health response to HIV [8]. These guidelines recommended that linkage to care be defined as the duration of time starting with HIV diagnosis and ending with enrolment in HIV care or treatment. However, this definition was ambiguous as to what data could be used to populate the linkage indicator and made no recommendations in what time period to use to define prompt linkage.

### Proposed definition

As part of the Second Health Programme, the European Commission co-funded the Optimising testing and linkage to care for HIV across Europe (OptTEST) project, with an aim to optimise testing and linkage to care for HIV in Europe through a series of work packages [9]. In September 2015, OptTEST hosted a workshop on standardising the linkage to care indicator at a wider expert meeting on the continuum of care hosted by the European Centre for Disease Prevention and Control (ECDC) [10]. The meeting was attended by 48 experts from 20 countries, including HIV surveillance leads from the European HIV Surveillance Network and representatives from European HIV patient cohorts. A full list of attendees can be found in the Supplement. Workshop discussions resulted in experts advocating for a pragmatic approach to monitoring linkage to care in Europe.

The WHO definition was endorsed and further operationalised, with linkage to care defined as patient entry into specialist HIV care after diagnosis, measured as the time between the HIV diagnosis date and either the first clinic attendance date, first CD4^+^ count or viral load date, or HIV treatment start date, depending on data availability. While, first clinic date after diagnosis was considered the gold standard marker for linkage to care, experts agreed that for many countries, routine baseline laboratory data, such as first CD4^+^ and viral load date, would be practical proxies for care entry. The WHO currently recommends CD4^+^ and viral load testing to be carried out at baseline for all newly diagnosed persons at the first clinic visit [11]. Treatment initiation was included as a marker for care to acknowledge the change in guidelines that state people diagnosed with HIV should start ART as soon as possible, regardless of CD4^+^ count, for optimal outcomes [12]. In addition, some countries in Europe with limited surveillance may not be able to collect bio-marker or clinic data but may have information on ART initiation linked to external funding for national treatment programmes. For countries with available data on multiple care indicators, first clinic date was preferred, followed by CD4^+^ count date, viral load measurement date and finally, treatment start date.

There was also discussion at the workshop around the accuracy of care indicator dates. It was recognised that the dates captured by surveillance systems across Europe vary and this may influence linkage calculations. Where possible, care attendance date should be captured as the date of first visit to the HIV specialist care provider, regardless of visit purpose. CD4^+^ and viral load dates should be the date that the patient blood sample was taken for testing at the specialist clinic. ART initiation date should reflect the clinic visit at which patients were first prescribed ART.

Prompt linkage to care was defined by experts as linkage to care within 3 months of diagnosis, in-line with guidance from the United States (US) Centers for Disease Prevention and Control (CDC) [13]. The decision to recommend a 3 month cut-off in Europe was based on research from the US showing that initiation of care within 3 months of HIV diagnosis is significantly associated with faster time to viral suppression [2]. Furthermore, data from the United Kingdom show that >95% of people diagnosed with HIV enter care within 3 months of diagnosis [14] and a 3 month cut-off has been used widely in the literature from Europe [7].

### Developmental approach

The expert-agreed definition of linkage to care utilises a number of markers to signify entry into HIV care, acknowledging that there are disparate models of care across Europe and data used to calculate linkage are captured to varying extents by different countries [15,16].

In 2016, a survey was developed as part of the OptTEST project, in collaboration with international experts from the ECDC, the WHO Regional Office for Europe, the HIV/AIDS Civil Society Forum, the EURO HIV EDAT (Operational knowledge to improve HIV early diagnosis and treatment among vulnerable groups in Europe) project, the European AIDS Treatment Group, and national experts from Public Health England and AIDS Fondet. The 30 national surveillance contact points for HIV in the European Union/European Economic Area (EU/EEA) were identified and invited to take part. The main objectives were to assess variables collected at the national level that could be used to monitor linkage to care, identify data caveats and receive feedback on the proposed definition. National data on diagnoses of HIV made between 2010 and 2014 were requested, alongside the proportion of those diagnoses that had entry marker data available ever after diagnosis and within 3 months of diagnoses (e.g. the proportion of people with a viral load available).

Responses were received from 24 (80%) of 30 EU/EEA national contact points (Table). Nineteen countries provided data on the number of new HIV diagnoses with at least one marker indicating subsequent care. Four countries commented on data availability but did not, or were not able, to provide data. Taking into account both the submitted data and narrative responses, linkage to care should be able to be calculated using the time difference between diagnosis date and (i) care attendance date in seven countries (ii) CD4^+^ date in 16 countries, (iii) viral load date in 11 countries and (iv) treatment initiation in six countries. Five countries collected markers of linkage to care but not the marker date, allowing calculation of the proportion ever linked to care but not the timeliness of linkage. The number of new HIV diagnoses in 2014 has been included in Table to show the relative size of the epidemics in each country, in the absence of prevalence data.

**Table:** Number of new human immunodeficiency virus (HIV) diagnoses made in 2014 and data availability to monitor subsequent HIV care at a national level, European Union and European Economic Area countries, September 2016 (n = 24 countries)

Country	New HIV diagnoses in 2014^a^	Care attendance	CD4^+^ count	Viral load	ART initiation
Belgium	1,050	NC	NC	NC	NC
Croatia	92	√	√	√	√
Cyprus	56	x	√	√	√
Czech Republic	232	√	√	√	x
Denmark	256	x	√^b^	X	x
Estonia	291	x	√	X	x
Finland	181	x	√^b^	X	x
France	5,653	x	√	√	x
Germany	3,500	x	√	√	x
Greece	761	x	√	X	x
Ireland^c^	363	x	√^b^	√^b^	x
Italy	3,850	x	√	√	x
Latvia	347	x	√	X	x
Lithuania	141	x	√^b^	√^b^	√^b^
Luxembourg	74	√	√	√	√
Malta	40	√	√	√	x
The Netherlands	881	√	√	√	√
Norway	267	x	x	X	x
Poland	1,133	x	x	X	x
Portugal	1,109	x	√^b^	√^b^	x
Romania	825	√	√	√	√
Slovenia	49	x	√	X	x
Spain	4,140	x	√	X	x
UK	6,157	√	√	√	√

NC: Not complete (country responded to other parts of survey but did not complete data table); √: data available; x: no data available; UK: United Kingdom.

^a^ Data from ECDC [26].

^b^ No date information collected.

^c^ Data on ART initiation and CD4^+^ and viral load dates collected from 2015 onwards.

The majority of countries not able to provide attendance or treatment data, cited problems with either the variable not being collected at all (attendance date: n = 9; treatment start: n = 10) and/or data not being reported centrally (attendance date: n = 14; treatment start: n = 12). The most common reasons for difficulty in providing CD4^+^ information was incomplete reporting to national surveillance by clinicians (n = 8) and when data were provided, significant reporting delay (n = 3). Viral load was more difficult to report than CD4+ because of a lack of centralised data collection mechanisms (n = 8). There were a few countries that reported issues collecting longitudinal patient data after diagnosis as care data were either housed in a separate clinical cohort database rather than collected as part of national surveillance (n = 3) or there was no legal framework for collection (n = 6).

## Discussion and conclusions

The expert-agreed definition of linkage to care presented here provides a pragmatic approach to the public health monitoring of this key HIV indicator that is appropriate to the European context. Linkage to care should be defined as patient entry into specialist HIV care after diagnosis, more specifically, the time between the HIV diagnosis date and either the first clinic attendance date, first CD4^+^ count, viral load date, or HIV treatment start date, with prompt linkage measured within 3 months. The application of this definition by researchers and public health professionals alike when reporting surveillance or research data relating to linkage to care after HIV diagnosis will enable reliable comparisons across countries, studies, population groups and allow monitoring of changes in linkage to care over time. The identification of gaps in linkage to care will allow for better insight into the barriers that may currently be limiting access to HIV medical care across Europe.

In order to apply this definition, national public health agencies and institutions should ensure adequate capture of clinical data on HIV diagnosis and entry in to care, such as CD4^+^ cell count. Of the 24 countries who responded to the survey, 16 could adopt this definition given their current national HIV surveillance system. However, eight reported poor completion rates of care marker data and 14 reported issues with collecting data centrally, which may require additional resources and time. An analysis of European HIV surveillance data revealed that even CD4^+^ data, which was considered the most appropriate indicator of linkage to care by EU/EEA countries, was only complete for ca 46% of new HIV diagnoses in the WHO European Region reported to the ECDC/WHO [17].

To determine the extent to which this expert definition is already being used in Europe, a systematic review of the literature was carried out in February 2017 [18]. Results indicate that 24 studies used the agreed definition to calculate linkage to care. Of the 22 that presented estimates, only 14 used a 3 month cut-off for prompt linkage. However, this definition and cut-off has also been applied to US, Canadian and Australian data [19]. To explore barriers to national monitoring of linkage to care using this definition and better understand the context within which linkage occurs, OptTEST organised meetings in collaboration with local partners in Greece, Poland and Portugal, bringing together key stakeholders from government, public health, community organisations and HIV care centres. These meetings highlighted the variability in the way HIV care and HIV surveillance systems are structured in each country [20-22], which is reflected in the flexibility in care markers used in the expert-agreed linkage to care definition.

The ability to calculate linkage to care also depends on robust reporting of date of HIV diagnosis. Although in discussion at the expert workshop, the importance of capturing data on a patient’s first reactive test was highlighted, it was recognised that the most practical date of diagnosis is the date the laboratory sample was taken for confirmatory HIV testing.

It is important to acknowledge that the agreed definition presented here is most appropriate for monitoring linkage to care at a national or clinic level and potentially less applicable to local community testing facilities. Also, co-funded by the European Commission, the Euro HIV EDAT project defined linkage to care as: ‘entry into health care or follow-up by an HIV specialist or in an HIV-unit after a reactive or confirmatory HIV test at a community testing facility.’ [23] Researchers found that though this was the most practical definition, community testing organisations across Europe face problems obtaining reliable information on whether a patient was successfully linked to care because of confidentiality and data protection issues. Often reporting of linkage is informal and limited date information is collected [23,24].

In conclusion, adoption of the expert-agreed definitions of linkage to care and prompt linkage presented here is needed to ensure consistent monitoring of the equitable access to HIV care and treatment. Application of a standard definition will help to better to understand time trends and to identify and compare populations most at risk of not attending for HIV care after diagnosis across Europe. The ECDC and the WHO Regional Office for Europe should continue to work with European countries to improve reporting of linkage to care data for public health monitoring. In addition, collaboration between public health bodies and national HIV clinical cohorts should be strengthened to address gaps in care data availability [25].
